# Prediction of treatment benefit in high-dimensional cox models via gene signatures in randomized clinical trials

**DOI:** 10.1186/1745-6215-16-S2-O86

**Published:** 2015-11-16

**Authors:** Nils Ternès, Federico Rotolo, Georg Heinze, Stefan Michiels

**Affiliations:** 1 CESP, INSERM U1018, Paris-Sud Univ., Villejuif, France; 2 Gustave Roussy, Service de biostatistique et d’épidémiologie, Villejuif, France; 3 Center for Medical Statistics, Informatics and Intelligent Systems, Medical University of Vienna, Vienna, Austria

## Background

Stratified medicine seeks to identify gene signatures predicting whether a patient will benefit from a treatment. We evaluated several approaches to identify such signatures using high-dimensional Cox models in randomized clinical trials (RCT).

## Methods

We investigated four approaches: penalize biomarker main effects and biomarker-by-treatment interactions using a lasso penalty (*full-lasso*); control of main effects by principal components or ridge penalty, and lasso on interactions (*sPCA+lasso* or *ridge+lasso*); and ‘*modified covariates*’ in a penalized regression model (*Tian et al. 2014*). We performed simulations under null and alternative scenarios by varying the sample size *n*, number of biomarkers *H*, number of true main effects or treatment-modifiers, effect sizes and correlations. We proposed two novel measures of treatment effect prediction for gene signatures: a difference in C-indices and a Wald-based interaction statistic. We used gene expression data from a RCT of adjuvant chemotherapy in non-small cell lung cancer (*n*=133) for illustration.

## Results

When *n*=500 and *H*=20 or 100, methods performed similarly in null scenarios apart from the *full-lasso* that gives poor results in presence of main effects only. In alternative scenarios: the *ridge+lasso* and the *full-lasso* predicted well the treatment benefit for future patients; the *modified covariates* approach performed poorly when also main effects were present. More extensive simulation results will be presented. In the lung cancer trial, the *full-lasso* and the *ridge+lasso* selected a gene signature with four and seven treatment-modifiers.

## Conclusion

Preliminary results suggest that *ridge+lasso* and *full-lasso* are promising approaches in high-dimensional Cox models to predict the treatment benefit.

